# Reward and Efficacy Modulate the Rate of Anticipatory Pupil Dilation

**DOI:** 10.1111/psyp.14761

**Published:** 2025-01-10

**Authors:** Joshua O. Eayrs, Haya Serena Tobing, S. Tabitha Steendam, Nicoleta Prutean, Wim Notebaert, Jan R. Wiersema, Ruth M. Krebs, C. Nico Boehler

**Affiliations:** ^1^ School of Psychology Liverpool John Moores University Liverpool UK; ^2^ Department of Experimental Psychology Ghent University Ghent Belgium; ^3^ Faculty of Psychology and Educational Sciences KU Leuven Leuven Belgium; ^4^ Department of Experimental Clinical and Health Psychology Ghent University Ghent Belgium

**Keywords:** efficacy, effort, preparatory control, pupillometry, reward

## Abstract

Pupil size is a well‐established marker of cognitive effort, with greater efforts leading to larger pupils. This is particularly true for pupil size during task performance, whereas findings on anticipatory effort triggered by a cue stimulus are less consistent. For example, a recent report by Frömer et al. found that in a cued‐Stroop task, behavioral performance and electrophysiological markers of preparatory effort allocation were modulated by cued reward and ‘efficacy’ (the degree to which rewards depended on good performance), but pupil size did not show a comparable pattern. Here, we conceptually replicated this study, employing an alternative approach to the pupillometry analyses. In line with previous findings, we found no modulation of absolute pupil size in the cue‐to‐target interval. Instead, we observed a significant difference in the rate of pupil dilation in anticipation of the target: pupils dilated more rapidly for high‐reward trials in which rewards depended on good performance. This was followed by a significant difference in absolute pupil size within the first hundreds of milliseconds following Stroop stimulus onset, likely reflecting a lagging effect of anticipatory effort allocation. Finally, the slope of pupil dilation was significantly correlated with behavioral response times, and this association was strongest for the high‐reward, high‐efficacy trials, further supporting that the rate of anticipatory pupil dilation reflects anticipatory effort. We conclude that pupil size is modulated by anticipatory effort, but in a highly temporally‐specific manner, which is best reflected by the rate of dilation in the moments just prior to stimulus onset.

## Abstract 2

We found pupils dilate in the final moments before a target in a Stroop task. The rate of dilation was more rapid for trials where the participant anticipated a large reward which was performance‐contingent, and individual differences in the rate of dilation predicted behavioural performance. These results provide new insights into the nature of preparatory effort and resolve previous contradictory results regarding the role of preparatory effort in pupil dilation.

Cognitive control is necessary for the performance of almost any task, both in the lab and in daily life, but exercising control is effortful and therefore typically avoided unless adequate incentive is available (Westbrook and Braver 2015). According to the Expected Value of Control model (Shenhav, Botvinick, and Cohen 2013; Shenhav, Cohen, and Botvinick 2016), the intensity of effortful control allocated to a task depends on a computation of the relative reward associated with successful performance and the effort necessary to obtain it. Functional imaging studies established that computation of the expected value of control is reflected in the activity of the dopaminergic midbrain and anterior cingulate cortex (ACC), and the resulting allocation of effort is associated with response preparation and activation of attentional control structures (e.g., Krebs et al. 2012; Shenhav, Botvinick, and Cohen 2013; Shenhav, Cohen, and Botvinick 2016). Electrophysiological studies have demonstrated that cues indicating reward prospects evoke significantly larger P3 ERP components, the amplitude of which scales with reward value. Later, approaching the target stimulus onset, reward and anticipated task demands interact to determine neural indices of task preparation such as the contingent negative variation (CNV) and posterior parietal alpha power (Goldstein et al. 2006; Kostandyan et al. 2019; Schevernels et al. 2014; Van Den Berg et al. 2014).

Pupil dilation provides a sensitive measure of the level of cognitive effort invested in a task in the sense that more difficult tasks lead to larger dilations, provided participants engage accordingly (Hess and Polt 1964; Kahneman and Beatty 1966; Van Der Wel and Van Steenbergen 2018). In tasks that involve cognitive conflict, such as the Stroop or flanker tasks (Eriksen and Eriksen 1974; Stroop 1935), pupil dilations to conflicting stimuli are reliably larger than those for non‐conflicting stimuli, reflecting the increased need for effortful control (Rondeel et al. 2015; Van Der Wel and Van Steenbergen 2018). Furthermore, individual differences in pupil responses to conflict predict behavioral outcomes—individuals with greater stimulus‐evoked pupil dilations respond faster, make fewer errors, and have smaller conflict‐related performance costs (Rondeel et al. 2015; Unsworth and Miller 2023). Physiologically, pupil size is tightly linked to the release of norepinephrine from the Locus Coeruleus (LC), which receives substantial input from the ACC. The LC has widespread efferent connections throughout the cortex and is thought to modulate neural gain—increasing excitation sensitivity to task‐stimulus‐relevant neurons (Alnaes et al. 2014; Aston‐Jones and Cohen 2005; Ferguson and Cardin 2020; Gilzenrat et al. 2010; Murphy et al. 2014; Reimer et al. 2016; Unsworth and Robison 2017).

While stimulus‐evoked pupil responses reliably index the allocation of cognitive effort to a task, the modulation of the pupil in anticipation of a task is less straightforward. Cognitive control can be allocated to a task both reactively (e.g., to resolve attentional conflicts arising from an unexpected distractor stimulus) but also proactively to minimize the need for reactive adjustments (e.g., by adopting an attentional set which already excludes potential distractors; Braver 2012). Accordingly, pupil size has been shown to increase in anticipation of target stimuli following cues indicating that a reward can be earned for good performance. This effect has been observed in various experimental paradigms, such as visual discrimination (Boehler et al. 2011), pro/antisaccade tasks (Wang, Brien, and Munoz 2015; Wang et al. 2016), and simple reaction time tasks (Schneider et al. 2018). However, in contrast, recent investigations using more complex cognitive paradigms have found little or no evidence for reward‐motivated preparatory effort allocation reflected in pupil size. Kostandyan et al. (2019) used both pupillometry and EEG to investigate the effects of reward on allocation of control to a flanker task. Participants completed an arrow flanker task (Eriksen and Eriksen 1974) in which they could earn rewards for fast and accurate responses. Similar to previous experiments, the cue‐evoked CNV component in anticipation of the target stimulus was significantly larger, and participants' responses were significantly faster for rewarded than unrewarded trials. Furthermore, pupil dilation evoked by the target stimulus was significantly larger for rewarded than unrewarded trials. However, there was no evidence that pupil size was differentially modulated for reward trials prior to the target onset. Although, intriguingly, the reward effect on the target‐evoked pupil response emerged almost instantly after target onset, several hundreds of milliseconds earlier than the effect of flanker congruence. This was taken to suggest that reward did modulate anticipatory pupil size, but in a way that was tightly linked with the temporal onset of the target stimulus.

In a recent extension of this line of work directly testing the Expected Value of Control model, Frömer et al. (2021) found that participants' proactive effort allocation depended not only on the level of reward but also on the ‘performance efficacy’ they anticipated in the task. In their experiments, participants were first presented with a cue indicating the monetary reward (relatively large or small), which could be earned on that trial as well as whether that reward would be purely performance‐contingent (i.e., a correct and fast response yielded a reward) or if it would be random (i.e., not dependent upon performance). They found that cued reward and efficacy interacted to determine participants' performance as well as replicating the effects of reward on the P3 and CNV components reported previously (with predicted efficacy behaving similarly to predicted task difficulty): CNV amplitude was greatest for large‐reward trials with high efficacy, suggesting that participants allocated more effort to the task when they expected this to be more instrumental in earning the reward. However, similar to Kostandyan et al. (2019) pupil dilation following the cue was not significantly modulated by reward and was in fact significantly smaller for high‐efficacy trials. The authors speculated that in their paradigm, pupil size effects reflected increased levels of arousal due to uncertainty induced by the low‐efficacy condition, rather than preparatory effort. At the same time, Frömer et al. (2021) fitted a canonical pupil response function that peaks in the cue‐target interval, which may not be ideal for capturing preparatory processes that gear up toward target presentation.

For example, Unsworth and Miller (2023) recently reported that pupil size ‘ramps up’ prior to the onset of the stimulus in a Stroop paradigm. That is, pupil size gradually increased over the last few hundreds of milliseconds before the stimulus appeared, and the authors suggest that the *rate* of pupil dilation (i.e., the speed at which the pupil increases in size) may be a better index of the allocation of preparatory effort than absolute pupil size. Furthermore, they found that individual differences in working memory capacity, RT on incongruent Stroop trials and behavioral Stroop congruency effects were all correlated with pupil size slope: Participants with larger working memory capacity, faster RTs and smaller Stroop congruency effects all had relatively steeper increases in pupil size. In a second experiment, they showed that the ‘ramping’ was also steeper when participants were cued to anticipate a conflicting stimulus than when they expected a congruent stimulus. The authors concluded that individuals with greater capacity for cognitive control (as indexed by working memory capacity; see e.g., Eayrs and Lavie 2018, 2019; Engle and Kane 2003) are better able to prepare for conflict, and this preparation is indexed by the rate of pre‐stimulus pupil dilation. While Unsworth and Miller (2023) did not include any incentive or reward manipulation, another study from the same group (Unsworth, Miller, and Aghel 2022) observed similar increases in pre‐stimulus pupil dilation rates for a simple psychomotor vigilance task when participants were simply cued to ‘try hard’ on a subset of trials. Similarly, Tromp, Nieuwenhuis, and Murphy (2022) related the first temporal derivative (i.e., rate of change, or ‘ramping’) of anticipatory pupil size to the effects of simulated neural gain on Stroop task performance (although their task did not include pre‐cues, stimulus onset was still predictable, allowing preparatory effort allocation). Similar to Unsworth and Miller (2023), they found that steeper anticipatory pupil dilation was associated with faster responding (albeit, in their study, at the cost of increased Stroop interference), globally supporting its use as an index of anticipatory neural gain.

The lack of reward‐ or efficacy‐modulated effects on pupil size during the cue‐to‐target interval in the results of Frömer et al. (2021) and, to some extent, also in Kostandyan et al. (2019) may be explained by two key factors: First, Frömer et al. (2021) used cues with significantly greater information‐density, informing about both the reward and efficacy of each trial, whereas the other studies included only information about either difficulty (Boehler et al. 2011; Wang, Brien, and Munoz 2015; Wang et al. 2016) or reward (Kostandyan et al. 2019; Schneider et al. 2018) or simply provided a warning for the start of the trial (Unsworth and Miller 2023). Prior work has established that relatively long periods can be necessary for proactive allocation to be affected following reward cues in tasks involving cognitive conflict (Bugg and Smallwood 2016), possibly due to the time taken to compute the expected value of control. Relatedly, Unsworth and Miller (2023) used a variable cue‐to‐target delay, which may have caused participants to allocate cognitive effort across a wider time‐range in order to also be ready for particularly early target trials. Second, Frömer et al. (2021) and Kostandyan et al. (2019) did not measure the ‘ramping’ of pupil size in the cue‐to‐target interval, but rather the absolute pupil size (or of the modeled pupil response function). According to the results of Unsworth and Miller (2023), proactive allocation of effort results in a gradually ramping pupil size rather than a transient dilation (or sustained larger pupil). This pattern is evident in several figures from previous reports, wherein pupil size can be seen to increase over the course of a cue‐to‐target interval (e.g., Boehler et al. 2011; Kostandyan et al. 2019; Schneider et al. 2018), sometimes only starting to diverge very near or at the onset of the target stimulus (Kostandyan et al. 2019). Indeed, in animal studies it has been shown that activity of cortical noradrenergic neurons is better characterized by the first temporal derivative of pupil size than by its absolute size (Reimer et al. 2016). Furthermore, in humans, the first temporal derivative of pupil size during pre‐stimulus baseline has been shown to predict subsequent behavioral performance (Tromp, Nieuwenhuis, and Murphy 2022; Van Den Brink, Murphy, and Nieuwenhuis 2016).

Here, we sought to investigate the effects of expected reward and performance efficacy on proactive allocation of cognitive effort as indexed by both absolute pupil size before and after stimulus onset and by the ‘ramping’ of pupil size in the pre‐stimulus interval. We used a paradigm modeled on that of Frömer et al. (2021) and with an analysis approach informed by the findings of Kostandyan et al. (2019) and Unsworth and Miller (2023). If, as suggested by the expected value of control theory, effort is allocated in proportion to its expected value, and if, as suggested by Unsworth and Miller (2023), this proactive effort is characterized by a gradually ‘ramping’ pupil size, then we should expect that a larger reward should result in steeper pupil size increases. Further, this effect should be modulated by expected efficacy, wherein effort is only allocated in trials where performance is critical to securing the reward. Following the results of Kostandyan et al. (2019), the effect may also be observed in absolute pupil size in the early portion of the stimulus–response, reflecting a lagging effect of this preparatory allocation.

## 1 Method

### 1.1 Participants

Based on the average of the three samples tested by Frömer et al. (2021), a sample of 38 participants, recruited from the Ghent University student pool, took part in the experiment in exchange for a cash payment of 12 Euros, with a maximum possible additional bonus payment of 10 Euros. Participants provided informed consent prior to taking part in the study. The research was conducted according to the ethical rules presented in the General Ethics Protocol of the Faculty of Psychology and Educational Sciences of Ghent University. One participant was excluded due to excessive blink‐related artifacts, leading to more than 50% of their data being interpolated. The final sample consisted of 6 men and 31 women, with an average age of 22.14 years (SD = 2.7). A sensitivity analysis using MorePower6.0 (Campbell and Thompson 2012) suggested that this would provide 82% power to detect an effect size of *η*_p_^2^ = 0.2, comparable to those reported by Frömer et al. (2021) for the interaction of efficacy and reward on CNV amplitudes and RT with a standard 0.05 alpha threshold. While our primary aim was to test these between‐condition effects, we also report exploratory individual differences analyses based on those of Unsworth and Miller (2023); a post hoc power analysis suggests that our sample of *n* = 37 would have approximately 46% power to detect a correlation of *r* = 0.29 as reported by Unsworth and Miller (2023).

### 1.2 Stimuli and Procedure

**FIGURE 1:**
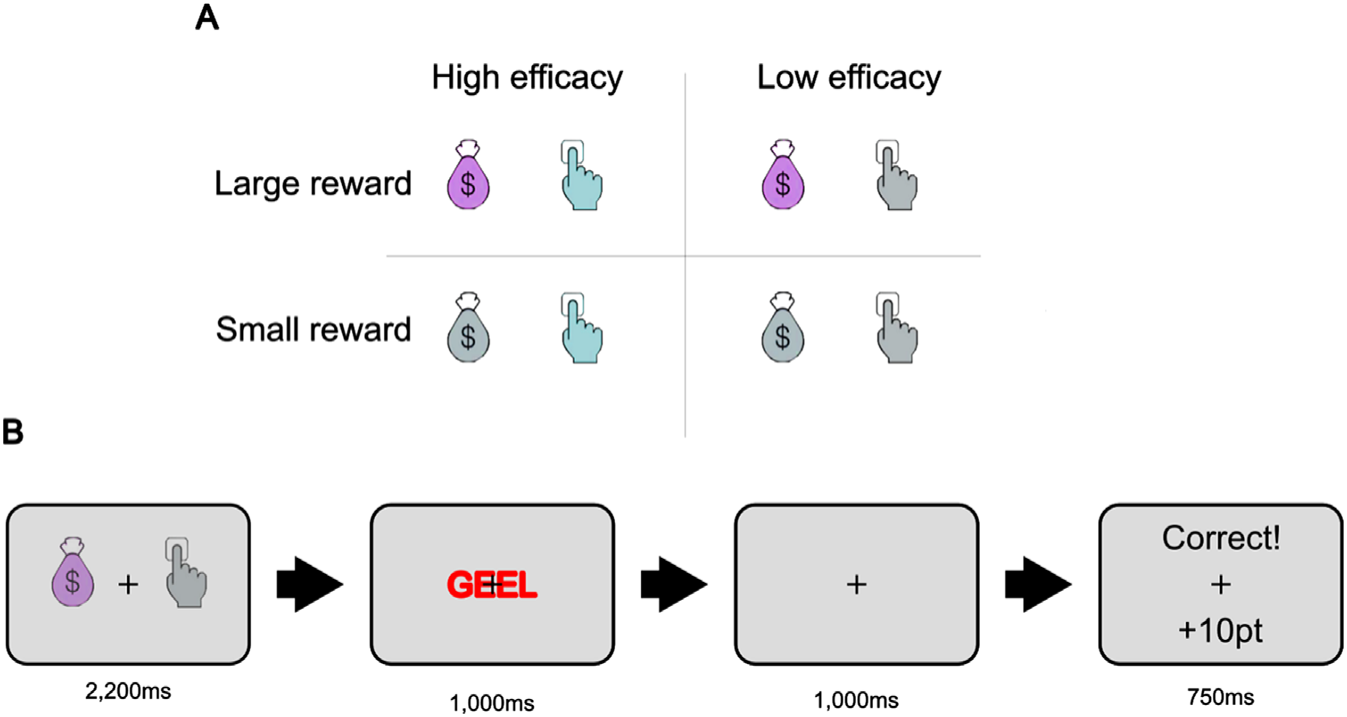
Experiment stimuli (A) and trial structure (B). For cue stimuli (A), gray always indicated ‘low’ reward and/or efficacy, while cyan and magenta ‘hands’ and ‘bags’ indicated ‘high’ reward and/or efficacy. The side of the screen on which the hand or bag was presented was constant for a given participant but counterbalanced between participants, as was the color coding for reward and efficacy. For each trial (B) all Stroop stimuli were incongruent (different word meaning and ink color; “GEEL” is the Dutch word for “YELLOW”); an ITI of 1500–2500 ms occurred between trials in which only the fixation cross was presented onscreen.

The task was based closely on the one used by Frömer et al. (2021). On each trial, participants were first presented with a cue stimulus (Figure 1A) consisting of two parts, an outline image of a hand and of a ‘money sack’; these images could either be gray or else colored in cyan or magenta to indicate the reward and efficacy condition. Isoluminance of the cue colors was confirmed with a Minolta LS‐110 luminance meter. Gray always indicated ‘low’ (reward or efficacy), whereas cyan and magenta indicated high (reward or efficacy; mapping counterbalanced between participants). The ‘hand’ and ‘sack’ component of the cue each subtended approximately 1.2 × 2.5 degrees of visual angle. Each was presented 1° to the left and right of the central fixation cross.

The cue remained onscreen for 2200 ms and was followed immediately by a Stroop stimulus consisting of the Dutch word for “Red” (“Rood”), “Yellow” (“Geel”), “Green” (“Groen”), or “Blue” (“Blauw”). The luminance of these colors was not controlled, but all colors appeared equally frequently in each reward and efficacy condition. The stimulus subtended between 3.4 × 2.5 and 4.5 × 2.5 degrees of visual angle and were presented at fixation. The Stroop stimulus remained on‐screen for 1000 ms before being replaced by a blank screen (fixation cross only) for a further 1000 ms. Participants were instructed to respond as fast and accurately as possible immediately upon presentation of the Stroop stimulus by pressing the “d”, “f”, “j”, or “k” key, each of which corresponded to a unique ink color (mapping counterbalanced between participants). A response deadline of 1000 ms determined whether their response was considered in‐time (in order to earn a reward on high‐efficacy trials, if the response was also correct). Finally, a feedback screen was presented, consisting of text indicating the reward earned (“0.1 pt” or “10 pt” for a correct and fast response in high or low reward, respectively; “0 pt” for an incorrect response and “Too slow!” for a response after the deadline). A fixation‐only ITI screen was presented between trials with a duration drawn randomly on each trial from a uniform distribution between 1500 and 2500 ms.

The reward policy on low‐efficacy trials was the same as that used by Frömer et al. (2021): Whereas for high‐efficacy trials, a correct response within the response deadline would be rewarded, for low‐efficacy trials, reward was not dependent upon participants' performance, but were instead drawn randomly from a rolling window of the past 10 high‐efficacy trials. That is, on each trial, a random sample was selected from the preceding 10 high‐efficacy trials and if this trial was rewarded then so was the present low‐efficacy trial. Thus, reward rates were approximately matched within a 10‐trial window between the high‐efficacy and low‐efficacy conditions. At the end of the experiment, 10 trials were drawn at random to determine the total bonus reward earned by the participant—which could therefore range between 0 and 10 Euros (mean = 5.17, SD = 1.25).

Before starting the main task, participants were required to complete several practice phases. First, they learned the response mapping by responding to the letters “XXXXX” presented at fixation in the same size, position, and colors as the Stroop stimulus. They were required to press the “d”, “f”, “j”, or “k” keys corresponding to the correct answer for each color as in the main task with no time limit. These trials were presented in blocks of 16 trials (with four repetitions of each ink color) and were required to achieve at least 70% accuracy in one block to proceed.

Next, participants were familiarized with the cue stimuli. The same cues as in the main task were presented on‐screen with text below displaying the possible meanings of the cue along with button assignments to indicate those options (e.g., “d: high reward, low efficacy”, “f: low reward, low efficacy” etc.). Participants pressed a key on the keyboard corresponding to the correct meaning of the cue, again with no time limit for responses. As in the response mapping phase, participants were required to achieve at least 70% accuracy for one block of 16 trials to proceed to the next phase. Next, participants practiced the primary task. This practice was identical to the experimental task, described above (as displayed in Figure 1). Once again, participants had to achieve at least 70% accuracy for one block of 48 trials in order to proceed to the main task.

Our task and procedure were thus identical to those used by Frömer et al. (2021) with the following changes: (1) Our cue‐to‐target interval was 700 ms longer (2200 ms instead of 1500 ms) to allow more time for preparatory pupil effects to emerge; (2) The luminance of our cue stimuli was matched in order to rule out differential luminance effects on pupil size; (3). We did not include congruent Stroop stimuli to ensure a consistently challenging task and that the cued efficacy and reward were the primary factors affecting performance; (4) The response deadline during the main task was 1000 ms instead of 750 ms to compensate for the increased RTs on incongruent trials.

### 1.3 Pupil Size Recording

Pupil size was recorded continuously throughout the main experimental task using an Eyelink 1000 Plus eyetracker (SR Research Ltd., Ottawa, ON, Canada). The camera was positioned below the computer monitor at a distance of 60 cm from the participant. Participants were instructed to remain still with their head in the chinrest throughout experimental blocks and to limit eye movements as much as possible. Data were recorded from both eyes where possible, with a sampling rate of 1000 Hz. However, due to experimenter error, the data for four participants were instead recorded at a sampling rate of 500 Hz, and so all other data were subsequently downsampled to this frequency. For three participants it was not possible to obtain a clear and reliable image of both eyes (e.g., due to the participants glasses), and so in these cases only the left eye was recorded.

### 1.4 Analysis

Mean accuracy and RT were computed for each participant in each condition of the main task and analyzed with repeated‐measures ANOVA (rANOVA). Trials with RTs faster than 200 ms or slower than the condition‐wise mean plus 2.5 SDs were excluded from further analysis.

**FIGURE 2:**
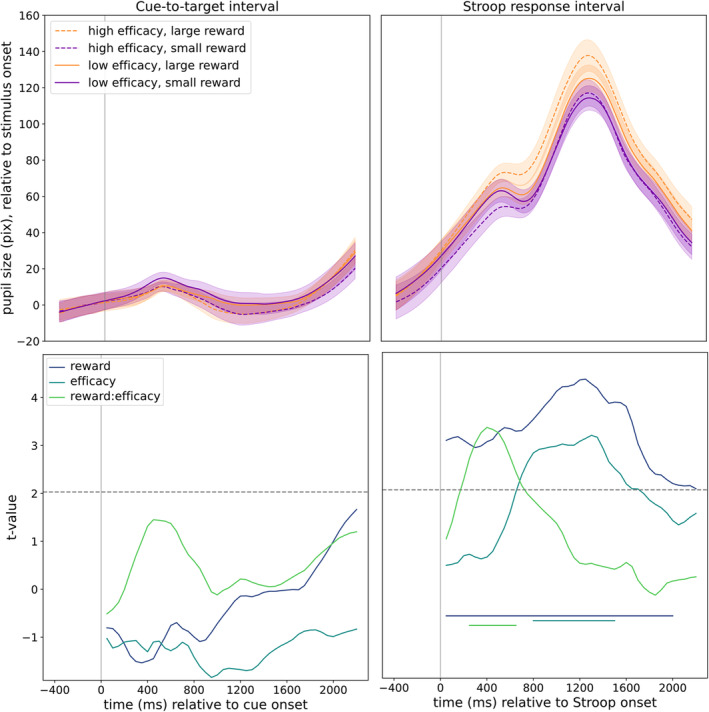
Mean, baseline‐corrected pupil size and *t*‐values for the post‐cue and post‐Stroop time‐windows of interest. Top left panel represents the mean, baseline‐corrected pupil size (pixels) during the cue‐to‐target interval; top right panel represents mean, baseline‐corrected pupil size following the Stroop stimulus; lower panels represent *t*‐value time‐course for comparisons of mean pupil size in sequential 50 ms bins for the cue‐to‐target interval (bottom left) and following the Stroop stimulus (bottom right). Solid vertical lines represent stimulus onset times; dashed, black horizontal lines indicate the *p* = 0.05 significance threshold; solid horizontal lines in the lower portion of the bottom right panel indicate *t*‐values which survive FDR correction for multiple comparisons.

Pupil data were analyzed in Python using custom scripts and some functions from the Pypillometry package (Mittner 2020). Data for each participant were preprocessed individually: First the data were smoothed using a Butterworth filter with a lowpass cutoff of 10 Hz. Next, blinks were identified based on velocity thresholds applied to the pupil size data (individual thresholds determined for each participant). Identified blink periods were then interpolated using a cubic spline as recommended by Mathôt et al. (2013). The complete, continuous pupil data for each participant were visually inspected in 5‐s epochs to ensure optimal detection and interpolation of blinks. Pupil data were then baseline‐corrected by subtracting the mean of the pupil size during the 500 ms preceding cue onset from the subsequent signal (for cue‐based analyses) or 500 ms preceding the Stroop target onset (for Stroop‐based analyses). Finally, event‐related pupil responses were calculated by taking the mean of all pupil traces for each condition (Figure 2). Mean, baseline‐corrected pupil size was then compared with repeated‐measures ANOVA (rANOVA) in sequential 50 ms bins throughout the cue‐to‐target and post‐Stroop intervals (in each case comprising 44 × 50 ms bins). The results of these analyses were then subject to a false‐discovery rate (FDR) correction for multiple comparisons. To analyze preparatory ‘ramping’ of pupil size, a linear function was fit to the mean absolute pupil size over the period from 1500 to 2500 ms after cue onset (with the baseline correction relative to cue onset as described above), thereby overlapping with the stimulus onset by 300 ms.

## 2 Results

### 2.1 Behavior

**FIGURE 3:**
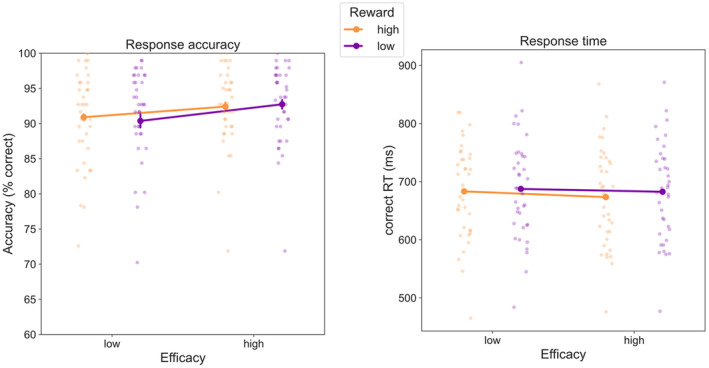
Mean response accuracy (left) and response times (right) in the Stroop task, error bars represent ±1 SE corrected for within subjects differences (Cousineau 2005).

Figure 3, left panel displays the mean correct reaction times for each condition. The corresponding rANOVA revealed a significant main effect of reward, *F*(1,36) = 5.88, *p* = 0.021, *η*_p_^2^ = 0.14, as participants responded faster to high‐reward trials. The main effect of efficacy was also significant, *F*(1,36) = 4.37, *p* = 0.044, *η*_p_^2^ = 0.11, with faster responses to high‐efficacy trials than low‐efficacy trials. Although numerically the fastest responses were for high‐reward and high‐efficacy trials, the interaction was not significant, *F*(1,36) = 0.60, *p* = 0.444, *η*_p_^2^ = 0.02.

A similar rANOVA applied to accuracy (Figure 3, right panel) did not reveal any fully significant effects for reward, *F*(1,36) = 0.03, *p* = 0.865, *η*_p_^2^ = < 0.01, efficacy, *F*(1,36) = 3.32, *p* = 0.077, *η*_p_^2^ = 0.08, or their interaction *F*(1,36) = 0.59, *p* = 0.447, *η*_p_^2^ = 0.02. Concerning the trend‐level main effect of efficacy, it is important to note that accuracy was higher for high efficacy, ruling out any speed‐accuracy trade‐offs (performance was authentically higher for high‐efficacy trials). Similarly, the absence of any numerical effect for reward also indicates that the RT benefit for these trials can be considered a real behavioral improvement.

### 2.2 Pupil Size

#### 2.2.1 Cue Response

Average pupil size was cut into sequential 50 ms bins, and the mean pupil size in each bin was analyzed in a repeated‐measures analysis of variance (rANOVA) with factors for reward (large, small) and efficacy (high, low) (Figure 2, left panels). There were no significant effects of reward, efficacy or their interaction at any point during the cue‐to‐target interval (all *F* < 2.49, all *p* > 0.123, uncorrected). These results therefore replicate the absence of cue‐evoked reward effects reported by Frömer et al. (2021) and others (Kostandyan et al. 2019); however, we also do not observe any effects of efficacy, contrasting with the larger pupils observed by Frömer et al. (2021) for low‐efficacy trials.

#### 2.2.2 Pre‐Stroop Slope

**FIGURE 4:**
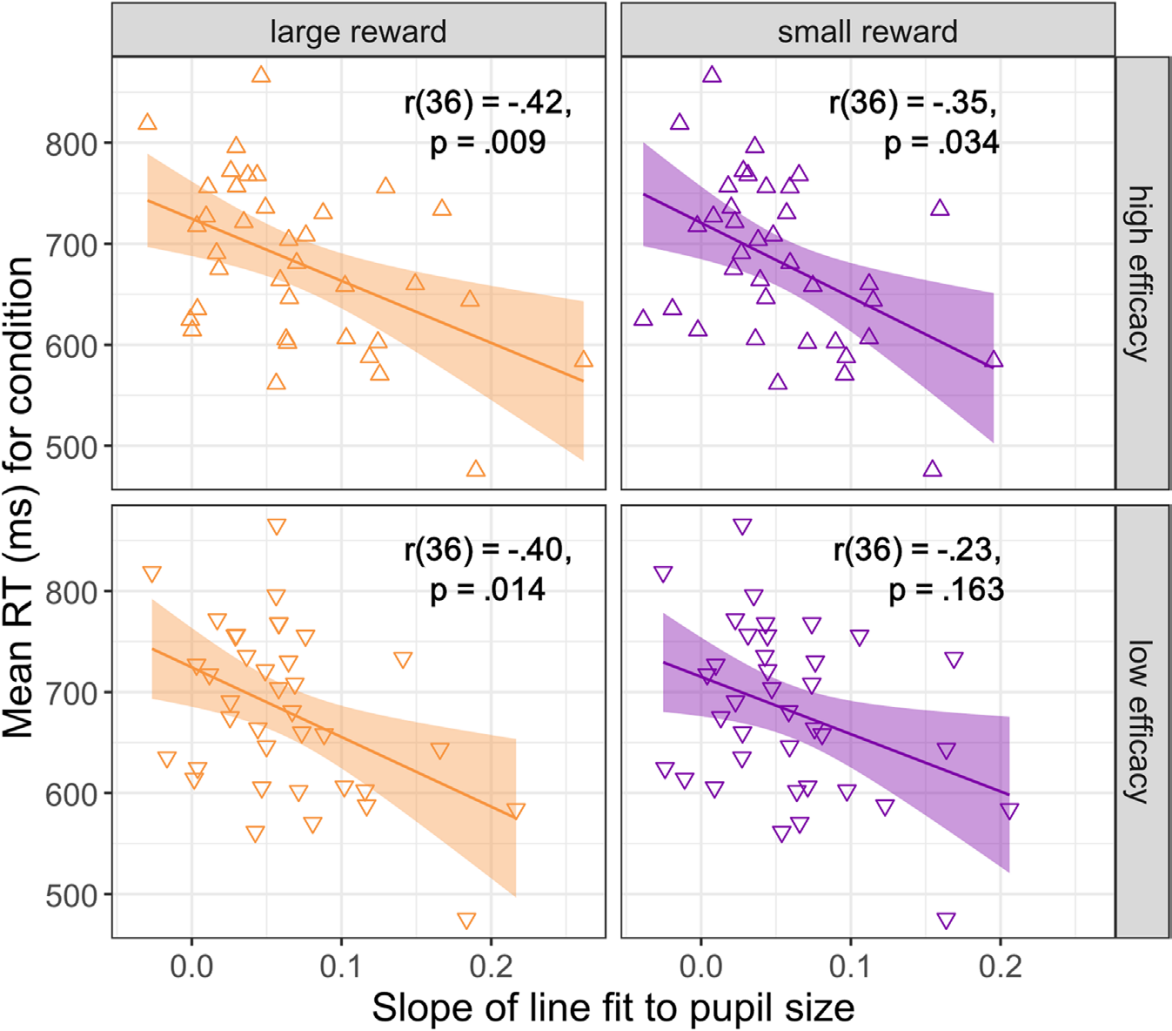
Scatterplots of the correlation between average RT and pupil dilation slopes for each experimental condition.

**FIGURE 5:**
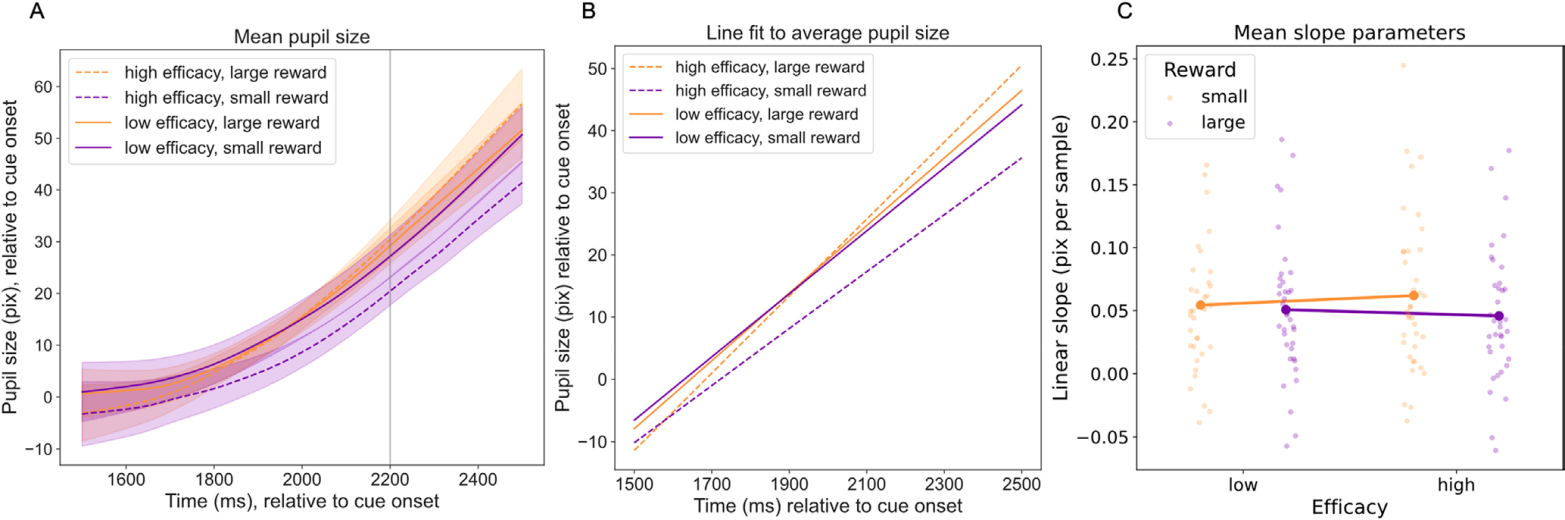
Average pupil size (A), linear fit to pupil size (B), and slope of the linear fits (C) within the time window from 700 ms before until 300 ms after the Stroop stimulus onset.

Our primary interest was in the effects of reward and efficacy on the rate of anticipatory pupil dilation (e.g., Unsworth and Miller 2023). To this end, a linear function was fit to the mean pupil size for each condition from 700 ms before Stroop onset until 300 ms after Stroop onset (Figure 4). We selected this time window to best capture the ‘ramping up’ effect in anticipation of the Stroop stimulus because, for all conditions, pupils began to dilate around 700 ms prior to target onset. We reasoned that Stroop‐stimulus‐evoked responses are unlikely to be observed until at least 400 ms or so after Stroop onset. The resulting linear slopes (Figure 5) were then compared via a rANOVA with factors for reward and efficacy.

This analysis revealed a significant effect of reward, *F*(1,36) = 13.8, *p* < 0.001, *η*_p_^2^ = 0.28, as slopes were significantly steeper for large than for small rewards. The main effect for efficacy was not significant, *F*(1,36) = 0.20, *p* = 0.656, *η*_p_^2^ < 0.01, but there was a significant interaction between reward and efficacy, *F*(1,36) = 6.18, *p* = 0.018, *η*_p_^2^ = 0.15. Follow‐up paired‐samples *t*‐tests revealed significantly steeper slopes (more rapid pupil dilation) for large rewards than for small rewards within the high‐efficacy condition, *t*(36) = 4.09, *p* < 0.001, *d* = 0.67 but not within the low‐efficacy condition, *t*(36) = 1.1, *p* = 0.279, *d* = 0.18.

#### 2.2.3 Stroop Response

As in the cue‐target interval, average pupil size was once again cut into 50 ms bins, and the average pupil size in each bin analyzed via rANOVA with factors for reward (large, small) and efficacy (high, low) (Figure 2, right panels), a FDR correction was applied to the resulting *p*‐values (surviving effects are represented by the solid horizontal lines at the top of the lower panel of Figure 2). As can be seen from Figure 2, a large and highly significant main effect of reward can be observed almost throughout the entire time window, as pupils were larger for large‐reward trials than for small‐reward trials. This effect is significant already within the first 50 ms time‐bin, *F*(1, 36) = 8.56, *p* = 0.006 (uncorrected, FDR‐corrected *p* = 0.019), *η*_p_^2^ = 0.19, suggesting that this reflects a late‐preparatory effect as opposed to a Stroop‐evoked response, which would take several hundreds of milliseconds to emerge (e.g., Kostandyan et al. 2019). The main effect of efficacy was not significant during this early time‐window, but became significant in later time bins, surviving FDR correction from 800 ms until 1550 ms (all *F* > 5.65, all FDR‐corrected *p* < 0.047). As can be seen from Figure 2, pupils were larger on high‐efficacy trials than on low‐efficacy trials. This main effect of efficacy was preceded by a significant interaction effect, which survived FDR correction in sequential bins from 250 ms until 650 ms (all *F* > 5.76, all FDR‐corrected *p* < 0.047). To better understand this interaction, we performed pairwise comparisons in each of the time‐windows where the interaction was significant. In each of these windows, pupils were significantly larger for high‐reward trials than for low‐reward trials, but only when efficacy was high (all *t*'s > 3.53, all *p*'s < 0.002) and not when efficacy was low (all *t*'s < 1.25, all *p*'s > 0.23).

#### 2.2.4 Individual Differences

Unsworth and Miller (2023) found that the slope of anticipatory pupil dilation was predictive of individual differences in working memory capacity and Stroop task performance (individuals with steeper pupil size increases had smaller Stroop effects and faster average RTs). Our study was not designed to measure individual differences and thus lacks the sample size or additional measures (working memory capacity) used by Unsworth and Miller. Nevertheless, for completeness, we assessed the correlation between individual's pupil dilation slopes and RT in each condition of our experiment (Figure 4). As can be seen from Figure 4, faster RTs within each condition were associated with steeper increases in pupil size. This was confirmed by the presence of a significant negative correlation between RT and the slope of the linear fit to pupil size in all conditions except for low‐reward and low‐efficacy (Figure 4, bottom right panel). The strongest correlation was between slope and RT for large‐reward and high‐efficacy trials, a comparison of correlation strengths conducted with the COCOR package in R (Diedenhofen and Musch 2015) indicated that this association was significantly stronger than the weakest, non‐significant correlation between pupil slopes and RT for small‐reward, low‐efficacy trials, all *z* = 2.2, *p* = 0.024 (see Table S1 for full correlation matrix and table of comparisons). The relationship between pupil dilation rate and behavioral performance in this task thus appears to depend upon the motivational factors relating to the expected value of control. As a control analysis, we also examined the correlations between absolute pupil size in the post‐Stroop interval and behavioral RT. These analyses (see Supporting Information) revealed the same pattern of results as for preparatory pupil size slopes, suggesting that, to a large extent, the differences in pupil size following the Stroop stimulus can be attributed to changes in preparatory effort allocation.

## 3 Discussion

In the present study, we investigated how anticipated reward and efficacy influence preparatory effort using pupillometry. Our work thereby strongly builds on the work by Frömer et al. (2021), who spearheaded work in this domain in an EEG‐pupillometry set‐up but observed unexpected pupillometry results. Using a slightly adjusted version of the cued‐Stroop task developed by Frömer et al. (2021), we cued participants to expect either large or small rewards, which could either be dependent on their performance or on a weighted coin‐toss. We found no modulation of absolute pupil size in response to the cues or in the cue‐to‐target interval; however, we did observe significant differences in pupil size following Stroop target onset. This effect began already within the first few hundreds of milliseconds, too early to have been triggered by the targets themselves, as pupil size changes with a delay of several hundred milliseconds for cognitive effects (Denison, Parker, and Carrasco 2020; Hoeks and Levelt 1993; Kostandyan et al. 2019). The effect therefore likely represents a late (or even well‐timed) engagement of preparatory effort in response to the cue. We also observed that pupil size began to ‘ramp up’ rapidly in the final moments before Stroop onset, replicating recent demonstrations by Unsworth and Miller (2023) and Tromp, Nieuwenhuis, and Murphy (2022), who have emphasized the rate of change to be an important characteristic of the pupil response as opposed to its absolute size. Crucially, we found that the slope of this pre‐target pupil dilation was modulated by reward and efficacy, such that it was steepest for large‐reward trials in which the reward was performance‐contingent. Finally, the rate of pupil dilation was significantly correlated with behavioral response times across participants, especially in performance‐contingent, large‐reward trials.

Our study was closely based on that of Frömer et al. (2021), who used the same cued reward and efficacy manipulation and measured pupil size and EEG responses. Our results differ from those of Frömer et al. (2021) in several key ways, however. First, they observed a significant interaction between reward and efficacy in response times, whereas we observed significant main effects of both reward and efficacy but no interaction (although our participants' response times did numerically replicate the pattern of results observed by Frömer et al.). It is noteworthy, though, that this interaction was only significant in two of the three experiments reported by Frömer et al. (2021). It is possible that slight differences in our task drove this difference: to emphasize the cues in our study and to avoid any possible confounds relating to congruency and success rate, we did not include congruent Stroop trials (i.e., trials where the ink color matches the word), whereas Frömer et al. did; we also used a less strict response time cutoff of 1000 ms instead of 750 ms. We did so in order to account for the fact that responses on incongruent trials are slower than on congruent ones, but probably increased the deadline to an extent that made it easier for participants to respond in‐time. More crucially, although Frömer et al. (2021) observed the predicted interaction between efficacy and reward in their anticipatory ERP (CNV) results, the pattern of cue‐related pupil dilation was markedly divergent from both the results we report here and their own predictions. Specifically, they observed no modulation of pupil size whatsoever by reward but significantly smaller pupils for high‐efficacy trials. Similarly, we observed no effect of reward on absolute pupil size in the cue‐to‐target interval, however, we also failed to observe any effect of efficacy. We did, however, observe significant differences in pupil size very soon after the Stroop stimulus onset, including an interacting effect of reward and efficacy. We also observed significantly steeper pupil dilation in the final hundreds of milliseconds prior to large‐reward trials, which interacted with efficacy. Both effects were generally in line with the expectations and with the preparatory state implied by the CNV findings by Frömer et al. (2021). The pupil analyses reported by Frömer et al. (2021) employed a modeling approach in which they compared the peak of a canonical pupil response following stimulus onset. Based on our results, this peak was likely too early to optimally capture the very late‐preparatory response to the cue, thereby obscuring any potential effects that may have been present. Similarly, this approach would not be suited to capture the differences in dilation slopes that we observed in the final moments before Stroop onset.

These results confirm and extend recent findings suggesting that the ‘ramping up’ of pupil size in anticipation of a Stroop stimulus reflects anticipatory effort (Tromp, Nieuwenhuis, and Murphy 2022; Unsworth and Miller 2023). Specifically, Unsworth and Miller (2023) found that pupil size increased linearly over the second prior to stimulus onset and was steeper when participants were cued to expect an incongruent than a congruent stimulus. Unsworth and Miller did not include any reward manipulation in their study, and we did not include a congruence or difficulty manipulation, but the convergence of results nevertheless suggests that the rate of pupil dilation reflects anticipatory effort allocation. Whether motivated by greater necessity (in the case of anticipated incongruent stimuli) or by incentive (in the case of reward), pupils dilate more rapidly when more effort is allocated to the task. In addition to the steeper slopes on trials where greater effort was required, Unsworth and Miller also observed significant correlations between individuals' pupil dilation rates and their behavioral response times (as well as congruency difference scores and working memory capacity; see also Tromp, Nieuwenhuis, and Murphy 2022). While not intended as an individual differences study, our results further replicate these effects and extend them to the context of reward motivation: Not only were steeper slopes associated with faster response times in our data, but this association also depended on motivational factors, such that the relationship was strongest for large‐reward trials in which the reward depended on good performance, whereas the association was significantly weaker and non‐significant in small‐reward, low‐efficacy trials.

These results provide a resolution to the contradictory results in previous literature regarding pupil size as a marker of preparatory effort. While several studies have provided evidence that pre‐stimulus pupil size can index reward‐induced preparatory effort (Boehler et al. 2011; Wang, Brien, and Munoz 2015; Wang et al. 2016), others have found no effect of reward cues in the cue‐to‐target interval, but only after the target stimulus has appeared (Frömer et al. 2021; Kostandyan et al. 2019). As noted, however, these studies do observe differences in absolute or baseline‐corrected pupil size very shortly after the target appears (e.g., Kostandyan et al. 2019), which we also observed here. Our results provide a possible explanation for these differences: anticipatory effort is reflected in pupil size, but only shortly before the onset of the stimulus, and the rate of pupil dilation is a better marker of this effort than absolute pupil size. This makes sense in the context of neurophysiological studies, which suggest that the first temporal derivative (rate of change) of pupil size is more tightly linked to cortical norepinephrine levels and with less temporal lag (Pfeffer et al. 2022; Reimer et al. 2016; Tromp, Nieuwenhuis, and Murphy 2022). Interestingly, this is then very closely followed by significant effects of reward and, slightly later, an interaction between reward and efficacy in the data that is time‐locked to the target onset. This closely mirrors earlier findings by Kostandyan et al. (2019), who observed no reward effects after a cue but immediate effects after target presentation, which were also interpreted as most likely still reflecting the influence of the cues. In fact, given that pupil size is known to be a lagged indicator of the underlying neural processes, it may indicate that participants were able to time their effort investment very well, getting ready just before target presentation. This may then be visible earlier in the ramping response toward the end of the cue‐target interval and a little later in pupil size itself.

Absolute pupil size and the slope of pupil dilation are clearly closely related constructs, but are comparatively weakly correlated (van den Brink et al. 2016), and have been shown to some extent to reflect distinct neural correlates (e.g., Reimer et al. 2016). Given that we observe both changes in pre‐stimulus pupil slopes and very early post‐stimulus changes in absolute pupil size, it is impossible to know the degree to which these effects reflect distinct underlying processes. However, both observations are in line with a proactive account, as both emerge before reactive control can have emerged (Braver 2012).

It may be the case that in our experiment, a very strong proactive mode of control was not required for participants to perform well. Indeed, the task used a relatively relaxed response deadline, and our participants performed very well (i.e., accuracy was generally around 90%). It is therefore possible that a more demanding task, in particular in the sense of tighter time–pressure, would encourage participants to recruit proactive control to a greater extent, which may then manifest in greater differences in pupil dilation or even absolute size prior to stimulus onset. However, the original findings of Frömer et al. (2021) suggest that this still may not be the case. Their response deadline was 25% shorter than ours, yet they still found no reward‐related effects on absolute pupil size. Nevertheless, the degree to which proactive control is the optimal strategic choice in a task is an important consideration which may also play into discrepancies in the previous literature, some tasks may benefit more than others from proactive control, and indeed some participants may have greater capacity to allocate either proactive or reactive control to a given task.

The pattern we observe in pupil dilation is strikingly similar to the pattern observed previously in the CNV component in similar studies (e.g., Frömer et al. 2021; Schevernels et al. 2014), thereby providing converging evidence that they may relate to a similar psychological process, arguably preparatory effort. Specifically, this component is reliably modulated by both reward and other task factors, including difficulty and efficacy, with the main effect of reward emerging first, shortly followed by an interaction in the last moments before target onset. In contrast, these studies typically also report a significant effect of reward on earlier (P3) ERP responses to cue stimuli, which is not reflected in a relatively early, cue‐evoked pupil response at any time in the 2‐s interval following the cue (i.e., of the type originally modeled by Frömer et al. 2021). This lends support to the suggestion that evaluation of the cued reward and efficacy (or difficulty) represents a distinct neurocognitive process to the later allocation of cognitive effort in anticipation of the task. That is, participants first compute the expected value of control for performing the task, then recruit arousal systems in accordance with that evaluation. The selective modulation of pupil dilation by the latter process would suggest a norepinephrinergic mechanism of this resource allocation (Reimer et al. 2016; Unsworth and Robison 2017). Future research could elucidate this further by directly assessing the association between pupil dilation and other neural/electrophysiological markers of effort allocation, such as the CNV component.

In our results, as in Frömer et al. (2021), participants still allocated significant effort to the task even on low‐efficacy (and low‐reward) trials, as reflected by the relatively high average accuracy and fast RTs achieved on these trials. This could in part reflect switch‐costs associated with changing levels of control from one trial to the next, as reconfiguring control settings may itself be effortful (Eayrs et al. 2024; Kool et al. 2010). Participants may therefore be slow to adjust their control settings, but with longer time horizons these adjustments may become more pronounced (see e.g. Grahek et al. 2023; Kukkonen et al. 2023). Another possibility is that participants invest cognitive effort into the task for reasons other than the incentives indicated by the cue stimuli, such as a learned association between effort and reward (e.g., Clay et al. 2022; Frömer et al. 2021) or social desirability of good performance.

In our results, it is not possible to completely distinguish between changes in pupil size related to reward anticipation as opposed to effort or resource allocation. Participants in the high‐reward condition and high‐efficacy conditions may have greater confidence in anticipating a positive outcome from the trial, which may manifest in greater levels of arousal. This explanation seems unlikely, however, as in our task reward probability on low‐efficacy trials was continuously matched to the reward rate on high‐efficacy trials (as in Frömer et al. 2021). Furthermore, there was no change in pupil size during the early cue‐to‐target interval, when participants were informed about the reward condition (whereas reward‐related changes in EEG indices of cue processing have been reported in the related previous literature; e.g., Frömer et al. 2021; Kostandyan et al. 2019; Schevernels et al. 2014). From Figure 2, it is also evident that the reward‐ and efficacy‐related changes in pupil size have already dissipated by the time reward feedback appeared onscreen. The fact that all effects of both reward and efficacy on pupil size are tightly linked to the onset of the Stroop stimulus, and the absence of cue‐evoked and feedback‐preceding differences in pupil size, suggest that these effects reflect preparatorys recruitment of cognitive effort to perform the task. Much in line with such an interpretation, Unsworth, Miller, and Aghel (2022) found analogous results to ours (steeper pupil dilation in anticipation of the target stimulus) with a cue simply prompting participants to ‘try hard’ without any reward incentive.

In conclusion, our results suggest that pupil dilation is a sensitive marker of effort allocation, particularly in the moments immediately preceding a task. While absolute pupil size in response to cues did not vary significantly with reward or efficacy before the end of the cue‐target interval, the rate of pupil dilation before target onset was clearly modulated by these factors, especially when large rewards were contingent on performance. This finding aligns with and extends previous research, highlighting the importance of considering the temporal dynamics of pupil responses, rather than just static measures of pupil size. The observed correlations between pupil dilation rates and behavioral response times further underscore the link between anticipatory effort and task performance, particularly under conditions of high motivational stakes. Future research should continue to explore the neurophysiological mechanisms underlying these effects, potentially integrating other markers of cognitive effort such as the CNV component to provide a more comprehensive understanding of how reward and efficacy influence preparatory effort.

## Author Contributions

**Joshua O. Eayrs:** conceptualization, data curation, formal analysis, investigation, methodology, project administration, software, validation, visualization, writing – original draft, writing – review and editing. **Haya Serena Tobing:** conceptualization, data curation, formal analysis, investigation, methodology, project administration, writing – review and editing. **S. Tabitha Steendam:** conceptualization, writing – review and editing. **Nicoleta Prutean:** conceptualization, writing – review and editing. **Wim Notebaert:** conceptualization, funding acquisition, writing – review and editing. **Jan R. Wiersema:** conceptualization, funding acquisition, writing – review and editing. **Ruth M. Krebs:** conceptualization, funding acquisition, writing – review and editing. **C. Nico Boehler:** conceptualization, funding acquisition, methodology, project administration, supervision, writing – review and editing.

## Conflicts of Interest

The authors declare no conflicts of interest.

## Supporting information


Data S1.
**Figure S1.** Full correlation matrix of Pearson’s *r*‐values for the individual differences analyses. Asterisks indicate level of significance: *p < 0.05, **p < 0.01, ***p < 0.001.
**Table S1.** Full table of comparisons of correlation strengths of pupil slopes and response times for each condition, including each of the comparison methods computed by COCOR. Bold text signifies significant comparisons.

## Data Availability

The data and code pertaining to this research will be made available upon publication on the Open Science Framework (OSF; https://osf.io/9yjdh/).
