# Supplementary material for: Reward and Efficacy Modulate the Rate of Anticipatory Pupil Dilation
Source: Psychophysiology. 2025 Jan 10;62(1):e14761. doi: 10.1111/psyp.14761 (PMC11718623; doi:10.1111/psyp.14761)
Supplement: Supplementary file 1 — Data S1. Figure S1. Full correlation matrix of Pearson’s r‐values for the individual differences analyses. Asterisks indicate level of significance: *p < 0.05, **p < 0.01, ***p < 0.001. Table S1. Full table of comparisons of correlation strengths of pupil slopes and response times for each condition, including each of the comparison methods computed by COCOR. Bold text signifies significant comparisons. [file PSYP-62-e14761-s001.docx]

# Supplementary materials

**Temporal jitter of maximal pupil slopes**

Following a suggestion by an anonymous reviewer, we sought to test whether our results could be explained by differences in the trial-wise timing of maximal slope values. Specifically, if the timing of maximal slopes are systematically different in one condition than another, or if they are simply more jittered, then it is possible that positive and negative slopes cancel out in the across-trial average. We therefore tested whether the maximal slope value on an individual-trial level could replicate our reported results, and whether there were differences in the timing or timing variability of maximal slope values between conditions. We quantified the per-condition maximum slope by taking the pupil size on each trial, computing the analytic first temporal derivative of pupil size, smoothing with a rolling average of 100 adjacent timepoints and finding the maximal resulting value of this derivative signal on each trial. These trial-wise maximal slopes were then averaged for each condition and analysed via a repeated-measures ANOVA with factor for efficacy and reward, this analysis yielded no significant effects (all F < 0.4, all p > .5). However, pupil size is sensitive to various factors, such as time-on-task, which may introduce considerable additional noise to the individual trial-level effects (e.g. van den Brink et al. 2016). We also compared the temporal latency and the variability of the temporal latency of trialwise maximal slopes, which also revealed no significant differences, suggesting that there is no systematic difference in the timing of pupil dilation or in the variability of this timing (see supplementary materials). As such, we think that our original results represent the most reliable estimate of the pupil dynamics before target onset, and that they were not strongly affected by differential timing of these dynamics across the different conditions.

Reward, F(1, 36) = 0.39, p = .535

Efficacy, F(1,36) < 0.01, p = .983

Interaction, F(1,36) = 0.04, p = .847

**Trialwise maximal slope times**

To test the possibility that the timing of maximal slopes was different in one condition than another, we conducted a similar analysis on the timing of the maximal slope values derived in the above analysis. Once again, there were no significant effects (all F < 3.2, all p > .08).

Main effect of Reward, F(1,36) = 0.15, p = .699

Main effect of Efficacy, F(1,36) = 3.13, p = .085

Interaction, F(1,36) = 1.022, p = .319

Finally, we conducted an analysis on the variability of the maximal slope times, which we quantified both by taking the standard deviation of maximal slope times and the average distance of each maximal slope from the median maximal slope. Both of these analyses also failed to reveal any significant effects.

**SD of trialwise maximal slope times**

Main effect of Reward, F(1,36) = 1.22, p = .277

Main effect of Efficacy, F(1,36) = 1.37, p = .249

Interaction, F(1,36) = 0.73, p = .397

**Average distance from median maximal slope time**

Main effect of Reward, F(1,36) = 3.23, p = .081

Main effect of Efficacy, F(1,36) = 0.96, p = .335

Interaction, F(1,36) = 0.12, p = .737

**Correlations with absolute pupil size**

Figure S1 presents the correlation matrix for behavioural RT, pre-stimulus pupil dilation slopes and absolute pupil size following the Stroop interval for each condition. The absolute pupil size was calculated as the mean pupil size from Stroop stimulus onset until 2,000 ms after, baseline corrected with respect to the 500 ms preceding Stroop stimulus onset (i.e. the entire window from time zero onwards, as depicted in the upper right portion of of figure 3). The correlations between absolute pupil size and RT are all strong and significant negative correlations, reflecting that larger pupil dilations were associated with faster RTs. The correlations between pupil slopes and absolute pupil dilations were all strongly positive and significant, reflecting that steeper pre-stimulus dilations were associated with larger pupil dilations following the Stroop stimulus. These results therefore support the conclusion that to a large degree stimulus-locked pupil dilations reflect a lagging indicator of anticipatory allocation of cognitive control, rather than purely reactive control.

# Figure S1. Full correlation matrix of Pearson’s r-values for the individual differences analyses. Asterisks indicate level of significance: * = p < .05, ** = p < .01, *** = p < .001

**
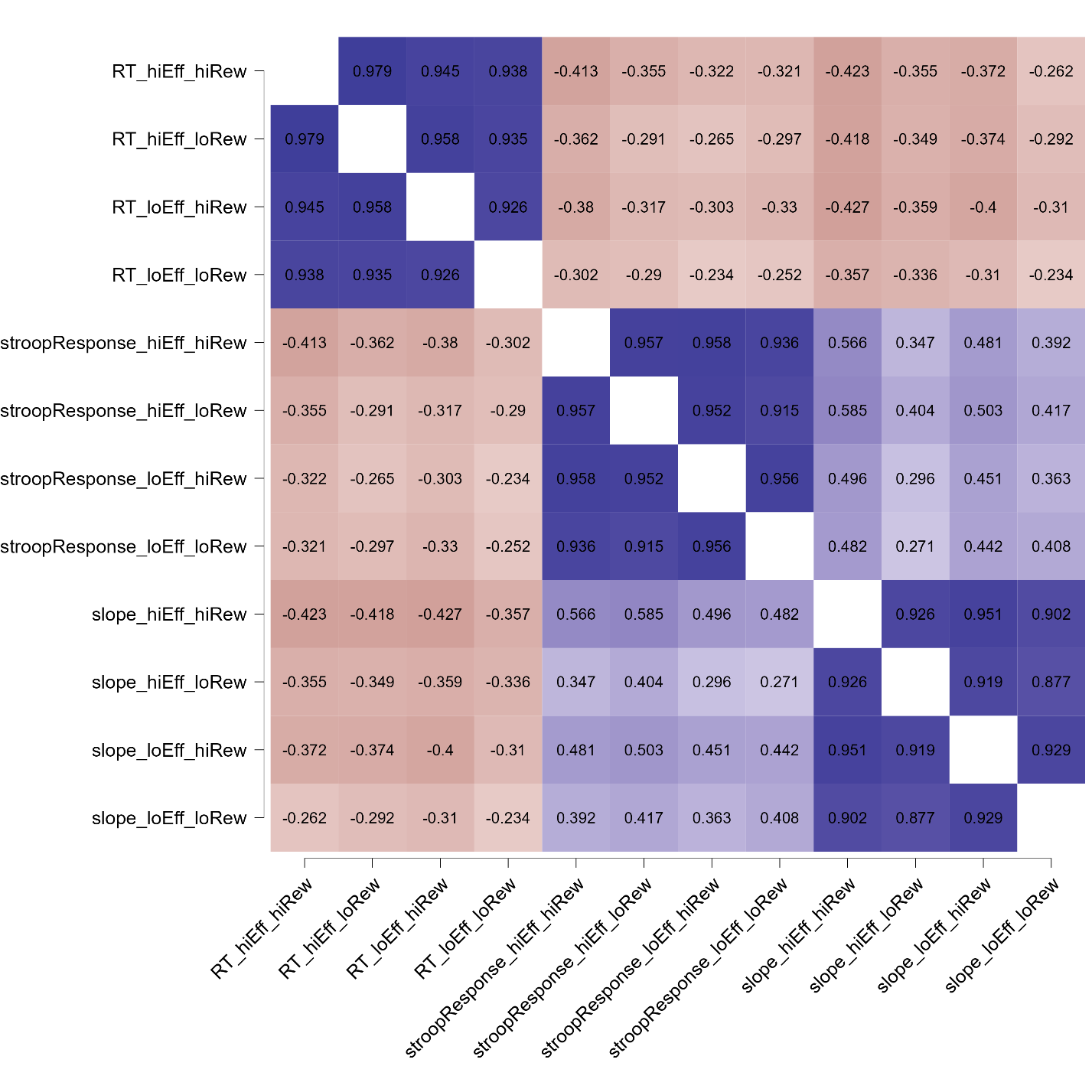
**

**Table S1.** Full table of comparisons of correlation strengths of pupil slopes and response times for each condition, including each of the comparison methods computed by COCOR. Bold text signifies significant comparisons.

|  | difference in r value | Pearson and Filon (1898) | Dunn & Clark (1969) | Steiger (1980) | Raghunathan et al. (1996) | Silver et al. (2004) | Zou (2007) | Conclusion |
| --- | --- | --- | --- | --- | --- | --- | --- | --- |
| high efficacy, high reward versus low efficacy, low reward | -0.19 | **z = -2.30, p = 0.021** | **z = -2.26,**  **p = .023** | **z = -2.19, p = .029** | **z = -2.26,**  **p = .024** | **z = -2.20,**  **p = .028** | **95% CI = [-.37, -.02]** | **Significant difference** |
| high efficacy, high reward versus high efficacy, low reward | -0.07 | z = -1.13, p = .26 | z = -1.9,  p = 0.276 | z = -1.08, p = .28 | z = -1.09,  p = .276 | z = -1.08,  p = .279 | 95% CI = [-.23, .07] | no difference |
| high efficacy, high reward versus low efficacy, high reward | -0.02 | z = -0.35, p = .721 | z = -0.34, p = .732 | z = -0.34, p = .732 | z = -0.34,  p = .732 | z = -0.34,  p = .732 | 95% CI = [-.17, .12] | no difference |
| high efficacy, low reward versus low efficacy, high reward | 0.05 | z = 0.70, p = .482 | z = 0.68,  p = .499 | z = 0.67, p = .5 | z = 0.68,  p = .499 | z = 0.67,  p = .5 | 95% CI = [-.10, .21] | no difference |
| high efficacy, low reward versus low efficacy, low reward | -0.39 | z = -0.43, p = .666 | z = -0.41, p = .679 | z = -0.41, p = .679 | z = -0.41,  p = .679 | z = -0.41,  p = .679 | 95% CI = [-.23, .15] | no difference |
| low efficacy, high reward versus low efficacy, low reward | -0.17 | **z = -2.07, p = .038** | **z = -2.03, p = .043** | **z = -1.97, p = .049** | **z = -2.03,**  **p = .043** | **z = -1.98,**  **p = .048** | **95% CI = [-34, -.004]** | **just significant difference** |
